# Quality of randomized controlled trials and systematic reviews in pediatric surgery: A cross‐sectional meta‐research study

**DOI:** 10.1002/cesm.12042

**Published:** 2024-02-04

**Authors:** Wilson Jiang, Bill Wang, Sandro Sperandei, Aidan Christopher Tan

**Affiliations:** ^1^ School of Medicine Western Sydney University Sydney Australia; ^2^ Translational Health and Research Institute Western Sydney University Sydney Australia; ^3^ NHMRC Clinical Trials Centre University of Sydney Sydney Australia

**Keywords:** meta‐analyses, methodological quality, pediatric surgery, randomized controlled trials, reporting quality, risk of bias, systematic reviews

## Abstract

**Background:**

There are few randomized controlled trials (RCTs) in pediatric surgery, and their risk of bias is unknown. There is also little known about the methodological or reporting quality of systematic reviews (with or without meta‐analyses) in pediatric surgery. Therefore, we conducted a cross‐sectional meta‐research study to determine the risk of bias and reporting quality of RCTs and systematic reviews and meta‐analyses in pediatric surgery, and the associations between these outcomes and study characteristics.

**Methods:**

We searched MEDLINE, Embase, Cochrane Library, JBI EBP Database, Centre for Reviews and Dissemination and Web of Science for all RCTs and systematic reviews in pediatric surgery published in 2021. We also searched the 2021 indexes of high‐impact pediatric surgery journals. We assessed the risk of bias and reporting quality of RCTs using the RoB 2 and CONSORT tools respectively. We assessed the same parameters for systematic reviews and meta‐analyses using the ROBIS and Preferred Reporting Items for Systematic Reviews and Meta‐analyses tools.

**Findings:**

We found 82 RCTs and 289 systematic reviews/meta‐analyses published in 2021. More than half (*n* = 46, 56%) of RCTs and almost all (n = 278, 96%) systematic reviews and meta‐analyses were at high risk of bias. Only one (1%) RCT and four (1%) systematic reviews and meta‐analyses were adequately reported. Less than half (*n* = 40, 49%) of RCTs and just over a quarter (*n* = 77, 27%) of systematic reviews and meta‐analyses had a registered protocol. Surprisingly, we found that more than half of systematic reviews and meta‐analyse (*n* = 162, 56.1%), had no risk of bias assessment.

**Conclusions:**

Recently published RCTs and systematic reviews in pediatric surgery are at high risk of bias and have poor reporting quality. Journals, universities, and research institutions should train authors to conduct and report higher quality studies and develop strategies to reduce risk of bias. However, research with high bias and low reporting does not necessarily lack value.

## 1 INTRODUCTION

### 1.1 Background

There are few randomized controlled trials (RCTs) in pediatric surgery. A review of 760 abstracts presented at the British Association of Pediatric Surgery Annual Congress between 1996 and 2000 found that only 1% (*n* = 9) were RCTs [1]. Similarly, a systematic review of studies in pediatric surgery published in 1998 and 2013 found that only 1.8% and 1.9%, respectively, were RCTs [2]. These figures are even poorer than those for adult surgery: a review of 87 surgical journals by Lombard et al. (2020) found that only 18% of surgical journals had published an RCT between 2016 and 2018 [3]. This is a long‐standing trend. RCTs declined from 14% of research articles in the British Journal of Surgery in 1985, to 5% in 1992 [4].

Barriers to conducting RCTs in pediatric surgery include inadequate funding [5], ethical concerns (e.g., issues with participant consent and concerns about impact on growth and development [6, 7]), recruitment challenges (e.g., relatively low incidence of many pediatric surgical conditions [8]), and conduct difficulties (e.g., difficulty in standardizing surgical procedures, varying experience levels of surgeons [4, 5]). Consequently, only 0.3%–11% of interventions in pediatric surgery are supported by well‐conducted RCTs, and more than 97% of clinical research in pediatric surgery consists of retrospective studies [5, 9], half of which are case series [8]. Therefore, it is imperative that these RCTs, and the systematic reviews which include them, are of high quality.

Risk of bias refers to the extent to which a study's design, conduct and analysis is likely to have prevented inaccuracies in the results [10, 11, 12]. Since risk of bias, and the overlapping concept of methodological quality, reflect the degree to which the study results can be trusted, RCTs and systematic reviews should ideally be at low risk of bias. A different concept is reporting quality, which refers to the extent to which a study provides a complete and accurate account of its design, conduct and analysis [10].

To the best of our knowledge, the risk of bias of RCTs in pediatric surgery has never been determined. There are also few studies on the methodological and reporting quality of systematic reviews in pediatric surgery, and the existing evidence, which suggests the quality is poor, is from over two decades ago [8, 13, 14, 15, 16, 17, 18, 19, 20].

### 1.2 Objectives

Our primary aim was to determine the risk of bias and reporting quality of recent RCTs and systematic reviews in pediatric surgery. Our secondary aim was to determine the study characteristics associated with risk of bias and reporting quality.

## 2 METHODS

### 2.1 Study design

This was a cross‐sectional meta‐research study of RCTs and systematic reviews in pediatric surgery published in 2021. In this manuscript, the term “systematic review” encompassed all systematic reviews, irrespective of whether their methodology included a meta‐analysis. The study protocol was prospectively registered on the Open Science Framework (https://osf.io/exmjp) and the study is reported in accordance with the Strengthening the Reporting of Observational studies in Epidemiology (STROBE) statement [21], and a reporting guideline for methodological research developed by Murad and Wang (2017), which was adapted from the Preferred Reporting Items for Systematic Reviews and Meta‐analyses (PRISMA) statement [22].

### 2.2 Setting

Studies were identified by searching the electronic bibliographic databases MEDLINE, Embase, Cochrane Library, JBI EBP Database, Centre for Reviews and Dissemination, and Web of Science (see Appendix A) on August 22, 2022. Additional studies were identified by searching the 2021 indexes of four high‐impact pediatric surgery journals (*Journal of Pediatric Surgery*, *Pediatric Surgery International*, the *European Journal of Pediatric Surgery*, and *Seminars in Pediatric Surgery*).

### 2.3 Studies

Studies were included if they met three inclusion criteria:

1 The authors described their study as either an RCT, systematic review or meta‐analysis. Authors who described their review as a narrative review, literature review or critical review, without reference to “systematic”, were not included.
2 The primary topic of the study related to pediatric surgery or an aspect or subspecialty of pediatric surgery. Studies which included both pediatric and adult populations were only included if more than half of study participants were aged under 18 years. Studies whose primary topic was intraoperative therapies which directly related to an invasive procedure (e.g., intraoperative imaging which directly altered immediate surgical management) were included. Studies whose primary topic related to noninvasive procedures (e.g., casting, shockwave lithotripsy, bracing, molding), postoperative therapies (e.g., tranexamic acid for postoperative bleeding, opioids and nerve blocks for postoperative pain, radiation as neoadjuvant therapy post tumor resection), fetal surgery, anesthetics, intensive care, dentistry, nursing or meta‐research were not included.
3 The study was published in full in 2021. Study protocols and studies written as replies, letters to the editor or secondary analyses were not included because their risk of bias and reporting quality could not be adequately assessed. Studies were not limited by language or location.

We downloaded all potentially relevant studies, uploaded them into EndNote 20 (Clarivate Analytics), removed duplicates and retrieved the full text of remaining articles. All articles written in English and Chinese were reviewed by a native speaker and those written in other languages were reviewed with the aid of Google Translate. Articles were screened for eligibility in duplicate by two independent investigators, and any discrepancies were resolved by discussion with a third investigator.

### 2.4 Variables

The following study characteristics were extracted from each article: year of publication, surname and affiliation of first author, number of authors, type of study, type of question, type of intervention, journal impact factor, journal or article PRISMA endorsement, registration and length of protocol, source of funding, conflicts of interest, centre status, sample size, range of participant age, types of studies included (for systematic reviews), number of studies included (for systematic reviews), number of data sources used, dropout rate, and crossover study status.

Definitions of these variables are reported in Appendix B.

### 2.5 Data sources/measurement

Included studies were assessed for risk of bias and reporting quality using existing tools. For RCTs, risk of bias was assessed with the RoB (Risk of Bias) 2 tool [23] and reporting quality was assessed with the CONSORT (CONsolidated Standards of Reporting Trials) 2010 statement [24]. The RoB 2 tool assesses risk of bias as low, some concerns or high across five domains and overall, and the CONSORT 2010 statement is a checklist of 37 items. For systematic reviews, risk of bias was assessed with the ROBIS (Risk Of Bias In Systematic reviews) tool [25], and reporting quality was assessed with the PRISMA 2020 statement [26]. The ROBIS tool assesses risk of bias as low, some concerns or high across four domains and overall, and the PRISMA 2020 statement is a checklist with 42 items. We defined adequate compliance to the CONSORT 2010 statement and PRISMA 2020 statements as reporting greater than or equal to 75% of items on the CONSORT checklist, in accordance with other studies [27, 28, 29, 30].

All included articles were assessed in duplicate by two independent investigators, and any discrepancies were resolved by discussion with a third investigator.

To further validate our risk of bias assessments, we attempted to compare our risk of bias assessments with those of other investigators. We searched the Web of Science database for all articles which cited our included studies, screened them for systematic reviews or meta‐analyses, and compared the risk of bias assessments in these studies with our own.

### 2.6 Bias

The methodological quality of our study was strengthened by duplicate screening and assessment of all articles by two independent investigators.

### 2.7 Study size

The study size was determined by the number of studies identified after the search and screening process.

### 2.8 Quantitative variables and statistical methods

Analyses were grouped into two categories based on study type: RCTs and systematic reviews/meta‐analyses. Results were presented as absolute and relative frequencies for categorical variables and as medians and interquartile ranges for continuous ones as they were not normally distributed.

The association between the study characteristics and risk of bias (low or some concern vs high) was assessed using two multivariate logistic regression models [31], one for RCT studies and other for systematic reviews. For the association with CONSORT score in RCT studies and PRISMA score in systematic reviews, multivariate linear regression models were built. A stepwise procedure was used with all models for variable selection. Results are presented in estimates and 95% confidence intervals. All analyses were performed in R, version 4.3.1. [32].

## 3 RESULTS

### 3.1 Studies

**Figure 1:**
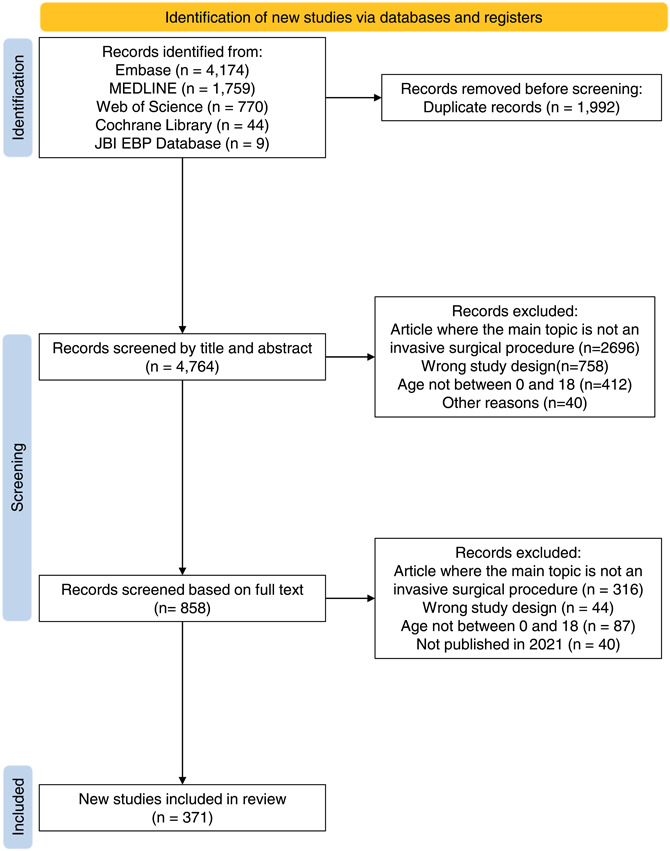
PRISMA flow diagram.

Figure 1 presents the PRISMA flow diagram of the search and selection process. We identified 6756 articles by searching electronic bibliographic databases and one additional article by searching the 2021 indexes of the four high‐impact pediatric surgery journals. We excluded 1992 articles after deduplication, 3906 articles after title and abstract screening and 644 articles after full‐text screening. We included a total of 82 RCTs and 289 systematic reviews or meta‐analyses published in 2021.

### 3.2 Descriptive data

The median journal impact factor was 1.9 (IQR = 1.1–2.7) for RCTs, and 2.4 (IQR = 1.8–3.5) for systematic reviews. The median number of authors was six (IQR = 5–8) for RCTs, and five (IQR = 4–7) for systematic reviews. For RCTs, the median loss to follow‐up rate was 7% (IQR = 2%–11%). Conflicts of interest were not declared in 14 (17.1%) RCTs, and 38 (13.1%) systematic reviews. The full list of studies and their characteristics are available in the supplementary data.

### 3.3 Outcome data

Only one (1%) RCT and four (1%) systematic reviews were adequately reported. For RCTs, the median number of CONSORT 2010 Statement items reported was 19/37 (IQR = 5–22), and for systematic reviews, the median number of PRISMA 2020 Statement items reported was 19/42 (IQR = 15–23). Less than half (40/82, 49%) of RCTs and just over a quarter (77/289, 27%) of systematic reviews had a registered protocol. The PRISMA and CONSORT items reported by more than 95% or fewer than 5% of studies are listed in Appendix D.

**Figure 2:**
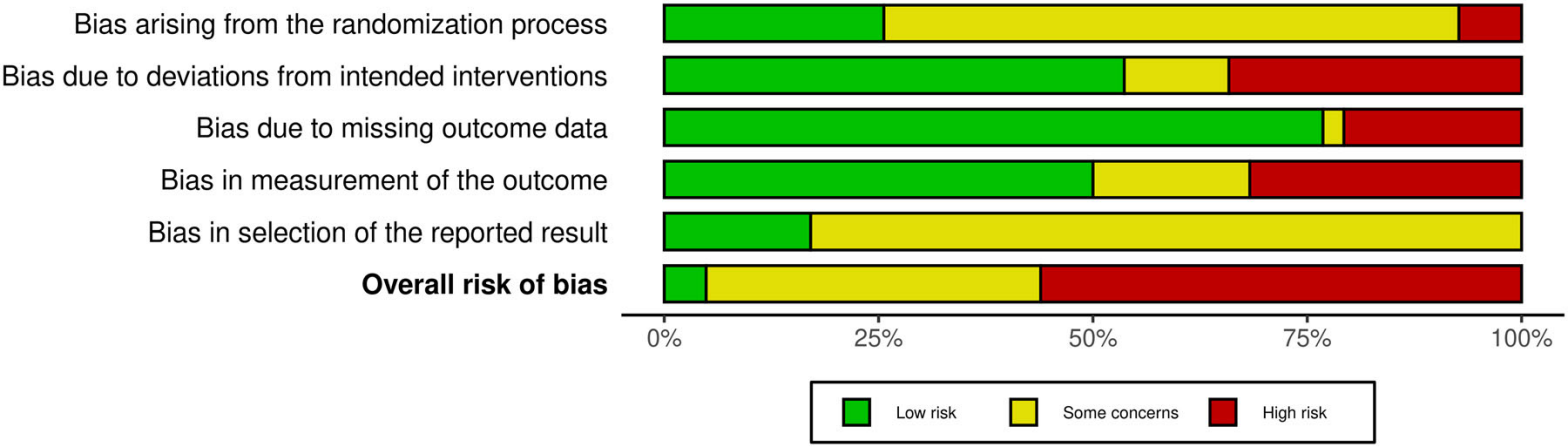
RoB 2 summary figure.

**Figure 3:**
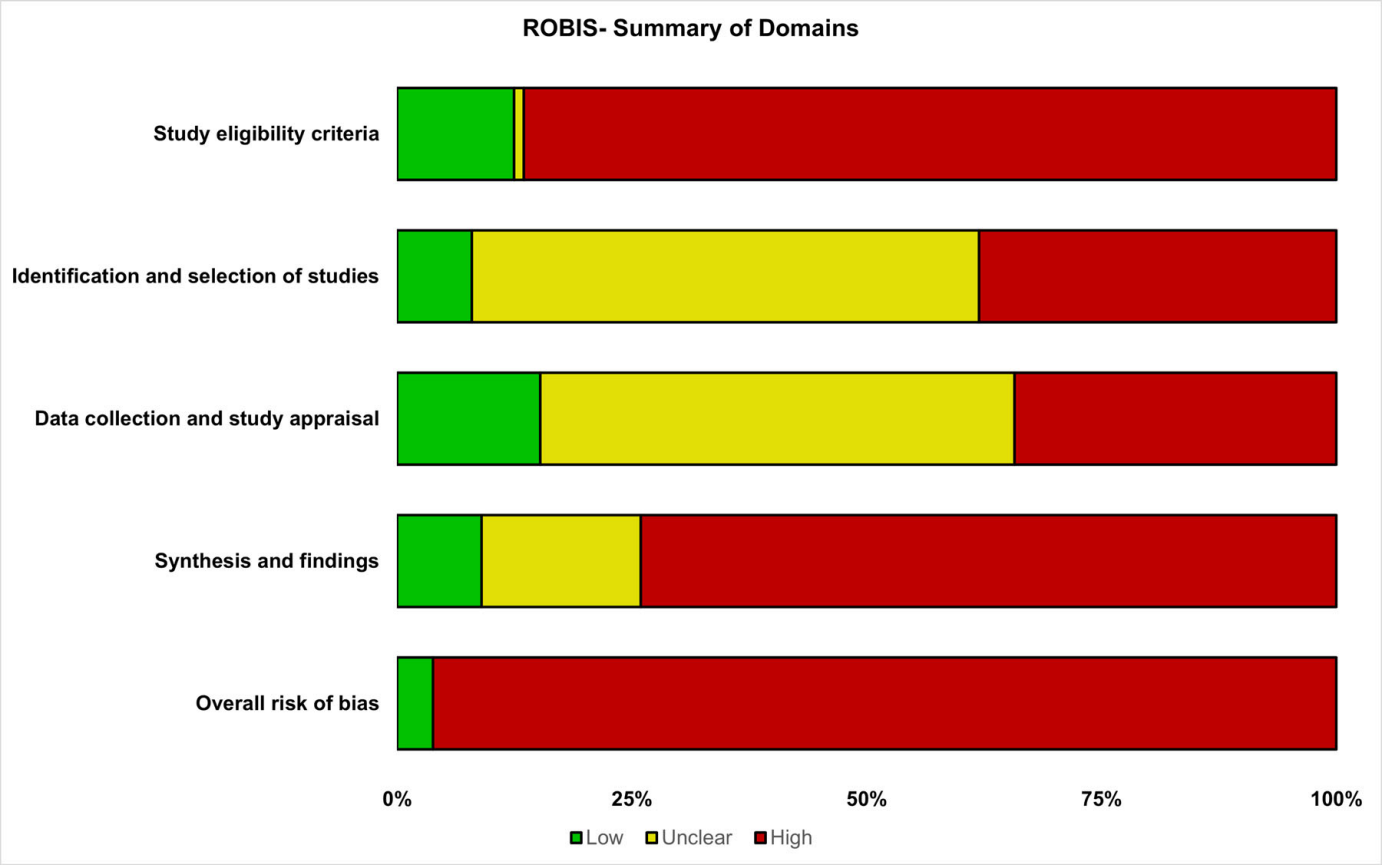
ROBIS summary figure.

More than half (46/82, 56%) of RCTs and almost all (278/289, 96%) systematic reviews were at high risk of bias. The risk of bias for RCTs and systematic reviews are summarized in Figures 2 and 3, respectively. In our attempt to further validate our risk of bias assessments, we did not identify any systematic reviews which both included the studies we included and assessed risk of bias using the same risk of bias assessment tools we used (see Appendix C).

The absolute and relative frequencies of items on the CONSORT 2010 and PRISMA checklists are presented in Table 1.

**Table 1:** CONSORT and PRISMA table.

CONSORT checklist item	*N* = 82, (*n* [%])
**Title and abstract**	
**1a. Title**	
Yes	52 (63.4%)
No	30 (36.6%)
**1b. Abstract**	
Yes	7 (8.5%)
No	75 (91.5%)
**Introduction**	
**2a. Scientific background and explanation of rationale**	
Yes	82 (100%)
No	0 (0%)
**2b. Specific objectives or hypotheses**	
Yes	82 (100%)
No	0 (0%)
**Methods**	
**3a. Description of trial design**	
Yes	37 (45.1%)
No	45 (54.9%)
**3b. Important changes to methods after trial commencement**	
Yes	7 (8.5%)
No	75 (91.5%)
**4a. Eligibility criteria for participants**	
Yes	82 (100%)
No	0 (0%)
**4b. Settings and locations where the data were collected**	
Yes	64 (78%)
No	18 (22%)
**5. Interventions**	
Yes	77 (93.9%)
No	5 (6.1%)
**6a. Completely defined pre‐specified primary and secondary outcome measures**	
Yes	18 (22%)
No	64 (78%)
**6b. Any changes to trial outcomes after the trial commenced, with reasons**	
Yes	0 (0%)
No	82 (100%)
**7a. How sample size was determined**	
Yes	40 (48.8%)
No	42 (51.2%)
**7b. When applicable, explanation of any interim analyses and stopping guidelines**	
Yes	7 (8.5%)
No	75 (91.5%)
**8a. Method used to generate the random allocation sequence**	
Yes	54 (65.9%)
No	28 (34.1%)
**8b. Type of randomization**	
Yes	32 (39%)
No	50 (61%)
**9. Allocation concealment mechanism**	
Yes	29 (35.4%)
No	53 (64.6%)
**10. Implementation**	
Yes	11 (13.4%)
No	71 (86.6%)
**11a. If done, who was blinded after assignment to interventions**	
Yes	49 (59.8%)
No	33 (40.2%)
**11b. If relevant, description of the similarity of interventions**	
Yes	8 (9.8%)
No	74 (90.2%)
**12a. Statistical methods used to compare groups for primary and secondary outcomes**	
Yes	79 (96.3%)
No	3 (3.7%)
**12b. Methods for additional analyses, such as subgroup analyses and adjusted analyses**	
Yes	17 (20.7%)
No	65 (79.3%)
**Results**	
**13a. Flow diagram**	
Yes	66 (80.5%)
No	16 (19.5%)
**13b. Losses and exclusions after randomization, together with reasons**	
Yes	32 (39%)
No	50 (61%)
**14a. Dates defining the periods of recruitment and follow‐up**	
Yes	43 (52.4%)
No	39 (47.6%)
**14b. Why the trial ended or was stopped**	
Yes	16 (19.5%)
No	66 (80.5%)
**15. Baseline data**	
Yes	69 (84.1%)
No	13 (15.9%)
**16. Numbers analysed**	
Yes	29 (35.4%)
No	53 (64.6%)
**17a. For each primary and secondary outcome, results for each group, and the estimated effect size and its precision (such as 95% confidence interval)**	
Yes	64 (78%)
No	18 (22%)
**17b. For binary outcomes, presentation of both absolute and relative effect sizes**	
Yes	8 (9.8%)
No	74 (90.2%)
**18. Results of any other analyses performed, including subgroup analyses and adjusted analyses**	
Yes	11 (13.4%)
No	71 (86.6%)
**19. All important harms or unintended effects in each group**	
Yes	60 (73.2%)
No	22 (26.8%)
**Discussion**	
**20. Limitations**	
Yes	75 (91.5%)
No	7 (8.5%)
**21. Generalizability**	
Yes	20 (24.4%)
No	62 (75.6%)
**22. Interpretation**	
Yes	80 (97.6%)
No	2 (2.4%)
**Other information**	
**23. Registration**	
Yes	38 (46.3%)
No	44 (53.7%)
**24. Protocol**	
Yes	37 (45.1%)
No	45 (54.9%)
**25. Funding**	
Yes	60 (73.2%)
No	22 (26.8%)

PRISMA checklist item	*N* = 289 (*n* (%))
**Title**	
**1. Title**	
Yes	288 (99.7%)
No	1 (0.3%)
**Abstract**	
**2. Structured summary**	
Yes	269 (93.1%)
No	20 (6.9%)
**Introduction**	
**3. Rationale**	
Yes	276 (95.5%)
No	13 (4.5%)
**4. Objectives**	
Yes	269 (100%)
No	20 (7.4%)
**Methods**	
**5. Eligibility criteria**	
Yes	276 (95.5%)
No	13 (4.5%)
**6. Information sources**	
Yes	137 (47.4%)
No	152 (52.6%)
**7. Search strategy**	
Yes	76 (26.3%)
No	213 (73.7%)
**8. Selection process**	
Yes	157 (54.3%)
No	132 (45.7%)
**9. Data collection process**	
Yes	138 (47.8%)
No	151 (52.2%)
**10a. Data items‐ define data outcomes**	
Yes	45 (15.6%)
No	244 (84.4%)
**10b. Data items‐ define study characteristics**	
Yes	5 (1.7%)
No	284 (98.3%)
**11. Study risk of bias assessment**	
Yes	127 (43.9%)
No	162 (56.1%)
**12. Effect measures**	
Yes	166 (57.4%)
No	122 (42.2%)
**13a. Effect measures‐ processes used to decide which studies were eligible for each synthesis**	
Yes	16 (5.5%)
No	272 (94.1%)
**13b. Effect measures‐ methods required to prepare the data for presentation or synthesis**	
Yes	60 (20.8%)
No	228 (78.9%)
**13c. Effect measures‐ methods used to tabulate or visually display results**	
Yes	69 (23.9%)
No	219 (75.8%)
**13d. Effect measures‐methods used to synthesize results**	
Yes	211 (73%)
No	77 (26.6%)
**13e. Effect measures‐ methods used to explore possible causes of heterogeneity among study results**	
Yes	34 (11.8%)
No	254 (87.9%)
**13f. Effect measures‐ sensitivity analysis**	
Yes	34 (11.8%)
No	254 (87.9%)
**14. Reporting bias assessment**	
Yes	76 (26.3%)
No	212 (73.4%)
**15. Certainty assessment**	
Yes	26 (9%)
No	262 (90.7%)
**Results**	
**16a. Study selection‐ PRISMA diagram**	
Yes	263 (91%)
No	25 (8.7%)
**16b. Study selection‐ cite excluded studies**	
Yes	14 (4.8%)
No	274 (94.8%)
**17. Study characteristics**	
Yes	261 (90.3%)
No	27 (9.3%)
**18. Risk of bias in studies**	
Yes	141 (48.8%)
No	147 (50.9%)
**19. Results of individual studies**	
Yes	259 (89.6%)
No	29 (10%)
**20a. Results of syntheses‐briefly summarize the characteristics and risk of bias**	
Yes	166 (57.4%)
No	122 (42.2%)
**20b. Results of syntheses‐Present results of all statistical syntheses conducted**	
Yes	198 (68.5%)
No	90 (31.1%)
**20c. Results of syntheses‐Present results of all investigations of possible causes of heterogeneity**	
Yes	26 (9%)
No	262 (90.7%)
**20d. Results of syntheses‐Present results of all sensitivity analyses**	
Yes	31 (10.7%)
No	257 (88.9%)
**21. Present assessments of risk of bias due to missing results**	
Yes	57 (19.7%)
No	231 (79.9%)
**22. Present assessments of certainty**	
Yes	20 (6.9%)
No	268 (92.7%)
**Discussion**	
**23a. Provide a general interpretation of the results**	
Yes	279 (96.5%)
No	9 (3.1%)
**23b. Discuss any limitations of the evidence included in the review**	
Yes	256 (88.6%)
No	32 (11.1%)
**23c. Discuss any limitations of the review processes used**	
Yes	74 (25.6%)
No	214 (74%)
**23d. Discuss implications of the results for practice, policy, and future research**.	
Yes	282 (97.6%)
No	6 (2.1%)
**Other information**	
**24a. Provide registration information for the review**	
Yes	87 (30.1%)
No	201 (69.6%)
**24b. Indicate where the review protocol can be accessed**	
Yes	77 (26.6%)
No	211 (73%)
**24c. Describe and explain any amendments to information provided at registration or in the protocol**.	
Yes	77 (26.6%)
No	211 (73%)
**25. Support**	
Yes	186 (64.4%)
No	102 (35.3%)
**26. Competing interests**	
Yes	250 (86.5%)
No	38 (13.1%)
**27. Availability of data, code and other materials**	
Yes	94 (32.5%)
No	194 (67.1%)

### 3.4 Main results

For RCTs, linear regression showed a positive correlation between a higher CONSORT score and the journal impact factor (*β* = .34, 95% CI = 0.190–0.484). The presence of a hospital‐affiliated first author was associated with a decrease in CONSORT score by 1.70 (95% CI = −3.325 to −0.066). RCTs with a published protocol had a CONSORT score that was 4.487 higher than those without a published protocol (95% CI = 2.920–6.053). Logistic regression showed that a lower risk of bias was correlated with a higher number of authors (OR = 0.844, 95% CI = 0.707–0.976).

For systematic reviews, a higher PRISMA score was associated with being a Cochrane review (*β* = 8.577, 95% CI = 3.782–13.373), having a published protocol (*β* = 5.885, 95% CI = 4.667–7.102), being unfunded (β = 1.213, 95% CI = 0.033–2.394), or government‐funded (*β* = 3.022, 95% CI = 0.828–5.176), and declaring a conflict of interest (*β* = 4.282, 95% CI = 1.757–6.807).A lower risk of bias was strongly associated with the presence of a published protocol (OR = 0.024, 95% CI = 0.002–0.154).

The statistical analyses are tabulated in Appendix E.

## 4 DISCUSSION

### 4.1 Key results

We conducted a cross‐sectional study of RCTs and systematic reviews in pediatric surgery published in 2021 and assessed their risk of bias and reporting quality. In general, risk of bias and reporting quality were suboptimal. Lower risk of bias and higher reporting quality were associated with the presence of a published protocol.

### 4.2 Interpretation

In RCTs, a larger number of authors was associated with lower risk of bias. This may be because collaboration and input from multiple experts contributes to addressing deficiencies in study design, data analysis, and reporting. As part of the research grant allocation process, allocators may wish to consider the proposed number of authors alongside other characteristics. The correlation between higher reporting quality and publication in higher impact factor journals is supported by previous evidence. A review of articles in all scientific fields by Thelwall et al. (2022) found a positive correlation between article quality and journal impact factor [33]. However, it's worth noting that Thelwall et al.'s study's findings may not be immediately applicable, given the very broad range of topics and study designs covered. While there may be a correlation between journal impact factor and higher reporting quality, all journals should require the same standard of methodological rigor from their authors.

In systematic reviews, higher reporting quality was associated with being a Cochrane review. This accords with a review of 141 systematic reviews in surgery by Yu et al. (2022), which found that methodological quality was positively associated with Cochrane review status [34] This finding is unsurprising as Cochrane is known for its rigorous methodological and reporting guidelines [35] and Cochrane reviews are widely considered as the gold standard for systematic reviews [36]. Journals should adopt stricter submission guidelines, following the example set by Cochrane. Notably, our findings revealed that unfunded and government‐funded reviews exhibited higher reporting quality compared to industry‐funded studies and those without declared funding, although the reasons for this observation are not immediately apparent. Previous studies have reported similar results, finding that industry‐funded systematic reviews may be associated with lower methodological quality in a number of topics, including vaccination,[37] drugs and medical devices [38] and asthma treatment [39]. Gøtzsche et al. (2012) proposed that the higher quality of nonindustry reviews was due to the larger number of co‐authors who are methodologists [40].

Intriguingly, systematic reviews that disclosed conflicts of interest demonstrated superior reporting quality compared to those which did not. While this relationship is at least partly influenced by conflict‐of‐interest reporting being part of the PRISMA criteria, authors who openly disclose conflicts of interest may exhibit greater transparency in other aspects of reporting, thereby elevating reporting quality.

In RCTs, systematic reviews, protocol registration was the most widespread predictor of quality, strongly correlating with reporting quality in RCTs and systematic reviews, and with risk of bias in systematic reviews. It is routine for journals to require a statement in the manuscript that the RCT is registered, along with the registration number [41]. However, a registration number was provided by fewer than half (38/82, 46%) of the included RCTs. This difference may relate to limited enforcement of trial registration by the journals. To enhance transparency and boost trial registration, journals should implement stricter reporting requirements for trial registration status.

It is noteworthy that many systematic reviews (162/289, 56%) were missing a risk of bias assessment. The findings of a systematic review depend heavily on the validity of its included studies [42]. Assessing the risk of bias of included studies is a critical step in the systematic review process and the absence of such assessments seriously undermines the utility of these systematic reviews. Authors of systematic reviews should evaluate the risk of bias in included studies, and journals should enforce stricter submission requirements for self‐identified systematic reviews, especially which do not appraise risk of bias. There may be a role for targeted education and training programs for systematic reviewers in pediatric surgery.

The risk of bias and reporting quality of RCTs, systematic reviews in pediatric surgery has not improved over the past two decades [5, 8, 13, 14, 15, 16, 17, 18, 19, 20] and risk of bias of RCTs in general has only modestly improved, and remains high [43]. Our findings are consistent with other studies in diverse disciplines such as anesthetics and surgery, which found that low quality and major flaws in RCTs are commonplace [43, 44, 45, 46, 47, 48].

Of interest are the clinical implications of our study results. It is generally agreed achieving statistical significance does not automatically translate to clinical significance [49, 50, 51, 52]. Clinical significance, which denotes an improvement in a patient's physical function, mental status, and/or quality of life [53], is a crucial consideration. Despite conducting an exhaustive literature search, we were unable to locate reliable evidence establishing a clear link between the use of guidelines based on biased evidence and poorer clinical outcomes. Consequently, considerable uncertainty exists regarding the influence of studies with a high risk of bias or low reporting quality on clinical outcomes in pediatric surgery. Until there is more widespread adoption of rigorous study designs and reporting standards, results of studies conducted in pediatric surgery should be taken with caution by meta‐researchers, guideline authors and clinicians alike.

This study exclusively addresses conclusions relating to risk of bias and reporting quality. However, there are practical constraints on research which naturally limit reporting quality or increase risk of bias. Such factors include ethical considerations [6, 7], challenges with patient recruitment [8], and the impossibility of blinding some interventions. Furthermore, the clinical applicability of a study's results is also dependent on factors outside of risk of bias and reporting quality, such as patient characteristics and external factors. As such, the actual overall clinical usefulness of each study's results may deviate significantly from the from the perceived poor quality suggested by the high risk of bias and low reporting quality assessments. We concur with the viewpoint presented in a commentary by Hamilton et al. (2023), which posits that research judged to be at a high risk of bias is not necessarily poor quality research [54].

### 4.3 Strengths and limitations

To the best of our knowledge, our sample size makes our study the largest review of reporting quality and risk of bias in pediatric surgery. Our study is the first to collect data on study characteristics and analyse for associations with reporting quality and risk of bias. This has allowed us to propose strategies to improve the quality of research in pediatric surgery.

However, our study has some limitations. The use of the RoB 2 tool, developed by Cochrane, to assess Cochrane reviews may have introduced a potential bias in our appraisal process. In this scenario, a potential concern arises because authors affiliated with Cochrane may have greater familiarity with the tool compared to authors from other institutions, where the RoB 2 tool might not be as widely recognized. This biases our assessment against publications from institutions or authors who have not yet acquainted themselves with RoB 2.

To improve the robustness of our conclusions, it would have been advantageous to search for articles published across a greater range of years. In the screening and data collection stages, the use of machine translation for non‐English studies may have introduced inaccuracies in interpreting study characteristics, methodology and outcomes. However, this issue would have had a limited impact as the proportion of articles which required translation was extremely small. Despite our exhaustive literature search, there may have been some studies which we missed.

### 4.4 Generalizability

As this study only reviewed RCTs, systematic reviews, the results presented in this paper are not generalizable to nonrandomised trials and observational studies. Although randomized controlled trials are widely regarded as the gold standard for evaluating the efficacy of clinical interventions, this dogma has been challenged by studies comparing treatment effect estimates in randomized versus nonrandomised trials [55, 56]. Future studies may wish to evaluate the quality of nonrandomised trials in pediatric surgery.

## 5 CONCLUSION

Recently published RCTs and systematic reviews in pediatric surgery are at high risk of bias and have poor reporting quality. We suggest strategies for how trialists, systematic reviewers and other stakeholders across the research lifecycle can design, conduct and report higher quality research in pediatric surgery.

## AUTHOR CONTRIBUTIONS

**Wilson Jiang**: Conceptualization; data curation; investigation; methodology; visualization; writing—original draft; writing—review and editing. **Bill Wang**: Conceptualization; data curation; investigation; methodology; resources; validation; writing—review and editing. **Sandro Sperandei**: Data curation; formal analysis; software; writing—review and editing. **Aidan Christopher Tan**: Conceptualization; data curation; investigation; methodology; project administration; resources; software; supervision; validation; visualization; writing—original draft; writing—review and editing.

## CONFLICT OF INTEREST STATEMENT

The authors declare no competing interests.

## 1 PEER REVIEW

The peer review history for this article is available at https://www.webofscience.com/api/gateway/wos/peer-review/10.1002/cesm.12042.

## Supporting information

Supplemental information.

Supplemental information.

## Data Availability

All data collected during the study will be available immediately following publication and with no end date, to anyone and for any purpose, on request to the corresponding author.

## APPENDIX A SEARCH STRATEGY

The keywords contained in the titles and abstracts of relevant articles identified by a pilot search, and the index terms used to describe these articles, were used to create a final search strategy with the assistance of an experienced medical librarian.

Database(s): **Ovid MEDLINE(R) ALL**

Search Strategy:

**Table:**

**#**	**Searches**	**Results**
1	Randomized controlled trials as topic/	157,540
2	Randomized controlled trial/	576,388
3	Random allocation/	106,877
4	Double blind method/	172,946
5	Single blind method/	32,168
6	Clinical trial/	536,049
7	Exp clinical trials as topic/	376,826
8	or/1–7	1,292,509
9	(clinic$ adj trial$1). tw.	447,184
10	((singl$ or doubl$ or treb$ or tripl$) adj (blind$3 or mask$3)). tw.	191,097
11	Placebos/	35,922
12	Placebo$. tw.	238,940
13	Randomly allocated. tw.	34,340
14	(allocated adj2 random). tw.	821
15	Or/9–14	740,449
16	8 or 15	1,624,670
17	Case report. tw.	371,551
18	Letter/	1,192,594
19	Historical article/	368,692
20	Review of reported cases. pt.	0
21	Review, multicase. pt.	0
22	Or/17–21	1,914,725
23	16 not 22	1,586,410
24	Systematic review. ti. or meta‐analysis. pt. or systematic literature review. ti. or this systematic review. tw, kf, hw. or pooling project. tw, kf, hw. or (systematic review. ti, ab. and review. pt.) or meta synthesis. ti. or meta‐analy*. ti. or integrative review. tw, kf, hw. or integrative research review. tw, kf, hw. or rapid review. tw, kf, hw. or umbrella review. tw, kf, hw. or consensus development conference. pt. or (1469‐493X or 1361‐6137). is.	382,316
25	(Clinical guideline and management). tw, kf, hw. or ((evidence based. ti. or exp evidence‐based medicine/or best practice*. ti. or evidence synthesis. ti, ab.) and (review or evaluation studies or validation studies). pt.)	37,372
26	(Systematic or systematically). tw, kf, hw. or critical. ti, ab. or study selection. tw, kf, hw. or ((predetermined or inclusion) and criteri*). tw, kf, hw. or exclusion criteri*. tw, kf, hw. or main outcome measures. tw, kf, hw. or standard of care. tw, kf, hw. or standards of care. tw, kf, hw.	1,706,987
27	(Survey or surveys). ti, ab. or overview*. tw, kf, hw. or review. ti, ab. or reviews. ti, ab. or search*. tw, kf, hw. or handsearch. tw, kf, hw. or analysis. ti. or critique. ti, ab. or appraisal. tw, kf, hw. or (reduction. tw, kf, hw. and (exp risk/or risk. tw, kf, hw.) and (exp “death”/or “death”. af. or (exp “recurrence”/or “recurrence”. af.)))	4,105,743
28	(Literature or articles or publications or publication or bibliography or bibliographies or published). ti, ab. or pooled data. tw, kf, hw. or unpublished. tw, kf, hw. or citation. tw, kf, hw. or citations. tw, kf, hw. or database. ti, ab. or internet. ti, ab. or textbooks. ti, ab. or references. tw, kf, hw. or scales. tw, kf, hw. or papers. tw, kf, hw. or datasets. tw, kf, hw. or trials. ti, ab. or meta‐analy*. tw, kf, hw. or (clinical and studies). ti, ab. or exp treatment outcome/or treatment outcome. tw, kf, hw.	4,340,214
29	(Letter or newspaper article). pt.	1,210,870
30	24 or 25	412,812
31	26 and 27 and 28	422,601
32	30 or 31	555,709
33	32 not 29	547,465
34	(Infant disease* or childhood disease* or neonatal disease* or (adolescen* or babies or baby or boy? or boyhood or girlhood or child* or girl? or infan* or juvenil* or neonat* or neo‐nat* or newborn* or new‐born* or pediatric* or peadiatric* or pediatric* or perinat* or preschool* or puber* or pubescen* or adolescen* or teen* or toddler? or youth*)). ti, ab, kf.	2,820,220
35	Exp adolescent/or exp child/or exp infant/	3,881,234
36	34 or 35	4,734,645
37	Exp “Specialties, Surgical”/or exp “Surgical Procedures, Operative”/or exp “Postoperative Complications”/	3,774,287
38	(“surg*“ or “postoperative” or “procedure” or “urolog*“ or “obstetrics” or “orthop*dics” or “ophthalmolog*“ or “neurosurg*“ or “otolaryngology” or “otorhinolaryngology” or “neurotology” or “surgical oncology” or “traumatology” or “Ambulatory Surgical Procedures” or “ablation” or “surgical anastomos*s” or “Anterior Temporal Lobectomy” or “Bariatric Surgery” or “Electrosurgery” or “Fasciotomy” or “Keratectomy” or “Laparotomy” or “Mastectomy” or “Metastasectomy” or “Microsurgery” or “Myotomy” or “Ostomy” or “Adenoidectomy” or “Laryngectomy” or “Laryngoplasty” or “Laryngoscopy” or “Cochlear Implantation” or “Endolymphatic Shunt” or “Mastoidectomy” or “Myringoplasty” or “Tympanoplasty” or “Pharyngectomy” or “Pharyngostomy” or “Tonsillectomy” or “Tracheo?tomy” or “Exenteration” or “Pneumonectomy” or “Splenectomy” or “Symphysiotomy” or “thyroidectomy”). ti, ab, kw.	3,235,012
39	37 or 38	5,380,771
40	33 and 36 and 39	18,357
41	limit 40 to yr = “2021”	1759

Database(s): **Embase Classic+Embase** 1947–2022 August 18

Search strategy:

**Table:**

#	Searches	Results
1	exp Meta Analysis/or ((meta adj analy$) or metaanalys$). tw. or (systematic adj (review$1 or overview$1)). tw.	512,798
2	(letter or editorial). pt.	1,971,133
3	Animal/	2,075,465
4	2 or 3	4,015,718
5	1 not 4	496,424
6	Clinical trial/or Randomized controlled trial/or Randomization/or Single blind procedure/or Double blind procedure/or Placebo/or Randomi?ed controlled trial$. tw. or Rct. tw. or Random allocation. tw. or Randomly allocated. tw. or Allocated randomly. tw. or (allocated adj2 random). tw. or Single blind$. tw. or Double blind$. tw. or ((treble or triple) adj blind$). tw. or Placebo$. tw.	1,967,827
7	Case study/or Case report. tw. or Abstract report/or letter/	1,853,964
8	6 not 7	1,919,792
9	5 or 8	2,263,547
10	(adolescen* or babies or baby or boy? or child* or girl? or infan* or juvenil* or juvenile* or neonat* or neo‐nata* or newborn* or new‐born* or pediatric* or peadiatric* or pediatric* or perinat* or preschool* or puber* or pubescen* or teen* or toddler? or youth*). ti, ab, kw.	3,757,942
11	exp newborn/or exp adolescence/or exp adolescent/or exp child/	4,260,275
12	10 or 11	5,324,975
13	exp surgery/or exp “surgery complication”/or exp “surgery coagulation”/or exp “surgery complications”/or exp “surgery ear nose throat”/or exp “surgery emergency”/or exp “surgery facial nerve”/or exp “surgery ICU”/or exp “surgery ICUs”/or exp “surgery intensive care unit”/	6,025,932
14	(“surg*“ or “postoperative” or “procedure” or “urolog*“ or “obstetrics” or “orthop*dics” or “ophthalmolog*“ or “neurosurg*“ or “otolaryngology” or “otorhinolaryngology” or “neurotology” or “surgical oncology” or “traumatology” or “Ambulatory Surgical Procedures” or “ablation” or “surgical anastomos*s” or “Anterior Temporal Lobectomy” or “Bariatric Surgery” or “Electrosurgery” or “Fasciotomy” or “Keratectomy” or “Laparotomy” or “Mastectomy” or “Metastasectomy” or “Microsurgery” or “Myotomy” or “Ostomy” or “Adenoidectomy” or “Laryngectomy” or “Laryngoplasty” or “Cochlear Implantation” or “Endolymphatic Shunt” or “Mastoidectomy” or “Myringoplasty” or “Tympanoplasty” or “Pharyngectomy” or “Pharyngostomy” or “Tonsillectomy” or “Tracheo?tomy” or “Exenteration” or “Pneumonectomy” or “Splenectomy” or “Symphysiotomy” or “thyroidectomy”). ti, ab, kw.	4,581,666
15	13 or 14	7,654,072
16	9 and 12 and 15	70,035
17	limit 16 to yr = “2021”	4174

Database(s): **JBI EBP Database** Current to August 31, 2022

Search strategy:

**Table:**

#	Searches	Results
1	((clinic$ adj trial$1) or ((singl$ or doubl$ or treb$ or tripl$) adj (blind$3 or mask$3)) or Placebo$ or “Randomly allocated” or (allocated adj2 random)). tw. or (“systematic review”. ti, ab. or meta‐analysis. pt. or meta‐analysis. ti, ab. or “systematic literature review”. ti. or “systematic review”. tw, hw. or “pooling project”. tw, hw. or (“systematic review”. ti, ab. and review. pt.) or “meta synthesis”. ti. or meta‐analy*. ti. or “integrative review”. tw, hw. or “integrative research review”. tw, hw. or “rapid review”. tw. or “umbrella review”. tw, hw. or “consensus development conference”. pt. or “practice guideline”. pt. or “drug class reviews”. ti.)	7294
2	(infant disease* or childhood disease* or neonatal disease* or adolescen* or babies or baby or boy? or boyhood or girlhood or child* or girl? or infan* or juvenil* or neonat* or neo‐nat* or newborn* or new‐born* or pediatric* or peadiatric* or pediatric* or perinat* or preschool* or puber* or pubescen* or adolescen* or teen* or toddler? or youth* or pediatric* or pediatric* or infan* or child* or adolescen*). ti, ab.	956
3	(“surg*“ or “postoperative” or “procedure” or “urolog*“ or “obstetrics” or “orthop*dics” or “ophthalmolog*“ or “neurosurg*“ or “otolaryngology” or “otorhinolaryngology” or “neurotology” or “surgical oncology” or “traumatology” or “Ambulatory Surgical Procedures” or “ablation” or “surgical anastomos*s” or “Anterior Temporal Lobectomy” or “Bariatric Surgery” or “Electrosurgery” or “Fasciotomy” or “Keratectomy” or “Laparotomy” or “Mastectomy” or “Metastasectomy” or “Microsurgery” or “Myotomy” or “Ostomy” or “Adenoidectomy” or “Laryngectomy” or “Laryngoplasty” or “Cochlear Implantation” or “Endolymphatic Shunt” or “Mastoidectomy” or “Myringoplasty” or “Tympanoplasty” or “Pharyngectomy” or “Pharyngostomy” or “Tonsillectomy” or “Tracheo?tomy” or “Exenteration” or “Pneumonectomy” or “Splenectomy” or “Symphysiotomy” or “thyroidectomy”). ti, ab, kw.	748
4	1 and 2 and 3	98
5	limit 4 to yr = “2021”	9

**Cochrane library**

**Table:**

#	Searches	Results
1	MeSH descriptor: [Child] explode all trees	61,756
2	MeSH descriptor: [Adolescent] explode all trees	110,478
3	MeSH descriptor: [Infant] explode all trees	35,072
4	MeSH descriptor: [Infant, Newborn] explode all trees	17,633
5	#1 or #2 or #3 or #4	160,486
6	(adolescen* or babies or baby or boy? or child* or girl? or infan* or juvenil* or juvenile* or kid? or neonat* or neo‐nata* or newborn* or new‐born* or pediatric* or peadiatric* or pediatric* or perinat* or preschool* or puber* or pubescen* or teen* or toddler? or youth*):ti, ab, kw	337,974
7	#5 or #6	337,974
8	MeSH descriptor: [Specialties, Surgical] explode all trees	2049
9	MeSH descriptor: [Surgical Procedures, Operative] explode all trees	129,429
10	MeSH descriptor: [Postoperative Complications] explode all trees	43,759
11	#8 or #9 or #10	142,382
12	(“surg*“ or “postoperative” or “procedure” or “urolog*“ or “obstetrics” or “orthop*dics” or “ophthalmolog*“ or “neurosurg*“ or “otolaryngology” or “otorhinolaryngology” or “neurotology” or “surgical oncology” or “traumatology” or “Ambulatory Surgical Procedures” or “ablation” or “surgical anastomos*s” or “Anterior Temporal Lobectomy” or “Bariatric Surgery” or “Electrosurgery” or “Fasciotomy” or “Keratectomy” or “Laparotomy” or “Mastectomy” or “Metastasectomy” or “Microsurgery” or “Myotomy” or “Ostomy” or “Adenoidectomy” or “Laryngectomy” or “Laryngoplasty” or “Cochlear Implantation” or “Endolymphatic Shunt” or “Mastoidectomy” or “Myringoplasty” or “Tympanoplasty” or “Pharyngectomy” or “Pharyngostomy” or “Tonsillectomy” or “Tracheo?tomy” or “Exenteration” or “Pneumonectomy” or “Reoperation” or “Splenectomy” or “Symphysiotomy” or “thyroidectomy”):ti, ab, kw	340,550
13	#11 or #12	402,354
14	#13 and #7	67,584
15	Restrict to 2021	44

**Web of science**

**Table:**

#	Searches	Results
1	TI = (clinic$ NEAR/1 trial$1) or TI= (Placebo$) or TI = (Randomly allocated) or TI = (allocated NEAR/2 random) or TI = (systematic review) or TI = (meta‐analysis) or TI = (systematic literature review) or TI = (systematic review) or TI = (pooling project) or TI = (meta synthesis) or TI = (meta‐analy*) or TI = (integrative review) or TI = (integrative research review) or TI = (umbrella review)	450,707
2	AB = (clinic$ NEAR/1 trial$1) or AB= (Placebo$) or AB = (Randomly allocated) or AB = (allocated NEAR/2 random) or AB = (systematic review) or AB = (meta‐analysis) or AB = (systematic literature review) or AB = (systematic review) or AB = (pooling project) or AB = (meta synthesis) or AB = (meta‐analy*) or AB = (integrative review) or AB = (integrative research review) or AB = (umbrella review)	412,243
3	1 or 2	673,024
4	TI = (infant disease* or childhood disease* or neonatal disease* or adolescen* or babies or baby or boy? or boyhood or girlhood or child* or girl? or infan* or juvenil* or neonat* or neo‐nat* or newborn* or new‐born* or pediatric* or peadiatric* or pediatric* or perinat* or preschool* or puber* or pubescen* or adolescen* or teen* or toddler? or youth* or pediatric* or pediatric* or infan* or child* or adolescen*)	2,264,148
5	AB = (infant disease* or childhood disease* or neonatal disease* or adolescen* or babies or baby or boy? or boyhood or girlhood or child* or girl? or infan* or juvenil* or neonat* or neo‐nat* or newborn* or new‐born* or pediatric* or peadiatric* or pediatric* or perinat* or preschool* or puber* or pubescen* or adolescen* or teen* or toddler? or youth* or pediatric* or pediatric* or infan* or child* or adolescen*)	2,236,335
6	4 or 5	3,364,232
7	TI = (surg* or “postoperative” or “procedure” or “urolog*“ or “obstetrics” or “orthop*dics” or “ophthalmolog*“ or “neurosurg*“ or “otolaryngology” or “otorhinolaryngology” or “neurotology” or “surgical oncology” or “traumatology” or “Ambulatory Surgical Procedures” or “ablation” or “surgical anastomos*s” or “Anterior Temporal Lobectomy” or “Bariatric Surgery” or “Electrosurgery” or “Fasciotomy” or “Keratectomy” or “Laparotomy” or “Mastectomy” or “Metastasectomy” or “Microsurgery” or “Myotomy” or “Ostomy” or “Adenoidectomy” or “Laryngectomy” or “Laryngoplasty” or “Cochlear Implantation” or “Endolymphatic Shunt” or “Mastoidectomy” or “Myringoplasty” or “Tympanoplasty” or “Pharyngectomy” or “Pharyngostomy” or “Tonsillectomy” or “Tracheo?tomy” or “Exenteration” or “Pneumonectomy” or “Reoperation” or “Splenectomy” or “Symphysiotomy” or “thyroidectomy”)	1,098,062
8	AB = (surg* or “postoperative” or “procedure” or “urolog*“ or “obstetrics” or “orthop*dics” or “ophthalmolog*“ or “neurosurg*“ or “otolaryngology” or “otorhinolaryngology” or “neurotology” or “surgical oncology” or “traumatology” or “Ambulatory Surgical Procedures” or “ablation” or “surgical anastomos*s” or “Anterior Temporal Lobectomy” or “Bariatric Surgery” or “Electrosurgery” or “Fasciotomy” or “Keratectomy” or “Laparotomy” or “Mastectomy” or “Metastasectomy” or “Microsurgery” or “Myotomy” or “Ostomy” or “Adenoidectomy” or “Laryngectomy” or “Laryngoplasty” or “Cochlear Implantation” or “Endolymphatic Shunt” or “Mastoidectomy” or “Myringoplasty” or “Tympanoplasty” or “Pharyngectomy” or “Pharyngostomy” or “Tonsillectomy” or “Tracheo?tomy” or “Exenteration” or “Pneumonectomy” or “Reoperation” or “Splenectomy” or “Symphysiotomy” or “thyroidectomy”)	2,930,420
9	7 or 8	3,494,914
10	9 and 6 and 3	7,023
11	limit 4 to yr = “2021”	770

## APPENDIX B DATA COLLECTION FORM

**Table:**

Study characteristics			
Year	—	—	—
Study type	What type of study	RCT	—
		SR	—
		MA	—
		SRMA	Labeled as both systematic review AND meta‐analysis and/or has features of both
First author name	—	—	—
Publication year	—	—	—
**Associations**			
First author affiliation	What type of institute the first author is a member of	Research institution	
		Hospital	
		University	
		Both	
		None	
Type of question	What is the main topic the researcher is trying to investigate	Intervention	Concerned with the effectiveness of a particular intervention aimed at preventing the development of a disease or a treatment, aimed at altering the course of a disease
		Prognosis	Determining the long‐term outcome of a surgical condition
		Diagnosis	Testing the sens, spec, PPV and/or NPV of a diagnostic surgical procedure
		Harm	
Type of review	Whether or not it's published in the Cochrane library	Cochrane	The Cochrane Library is a collection of systematic reviews, reviewed by a team of volunteer experts. The SRs published in Cochrane Library have been considered more up to date and of better quality than other reviews', and they incorporate “superior methodological rigor”
		Non‐Cochrane	
Journal impact factor	Scientometric index calculated by Clarivate that reflects the yearly mean number of citations of articles published in the last 2 years in a given journal, as indexed by Clarivate's Web of Science. As a journal‐level metric, it is frequently used as a proxy for the relative importance of a journal within its field; journals with higher impact factor values are given status of being more important, or carry more prestige in their respective fields, than those with lower values	—	
Journal or Article PRISMA endorsement		At least 1 endorses	PRISMA endorsement by journals is typically exemplified by a statement in the journal's Instructions to Authors indicating the journal's support of PRISMA
		Neither endorse	
Review registration	For SRMAs only	Yes	
		No	
Protocol registration	For RCTs only	Yes	
		No	
Length of protocol		—	
Number of authors		—	
Funding		Industry	Funded by a company for example, via sponsorship
		Government	
		Other	
		None	
Conflicts of interest		Present	A conflict of interest is clearly stated in the study
		Absent	The absence of COI is clearly stated in the study
		Unclear	The study does not clarify whether there's conflict of interest
Centre status		Single centre	
		Multicentre & single country	
		Multicentre and multi‐country	
Sample size		—	
Participant age (range)		—	
Types of studies included (for SRs & MAs only)		RCTs only	
		Nonrandomised trials only	
		RCTs and nonrandomised trials	
Type of intervention		Surgery only	
		Surgery + other intervention	
Number of studies included (for SRs & MAs only)		—	
Number of data sources used (SRs and MAs only)	Studies for an article may be extracted from (but not limited to): Medline, Embase, ISI of Knowledge, Database of Abstracts of Reviews Cochrane Central Register of Controlled Trials, Science Direct, Scopus,	—	
Dropout rate	Noncompletion of a study by a participant	—	
Crossover study (for RCTs and nonrandomised studies only)	A longitudinal study in which subjects receive a sequence of different treatments (or exposures)	Yes	
		No	
Lost to follow up rate (for RCTs and nonrandomised studies only)	The percentage of patients who at one point in time were actively participating in a clinical research trial, but have become lost (either by error in a computer tracking system or by being unreachable) at the point of follow‐up in the trial.	—	
Blinding (for RCTs and nonrandomised studies only)		Open label	Participants, doctors, researchers all know the treatment allocations
		Single blind	
		Double blind	

## APPENDIX C DATA VALIDATION

**Table:**

Reason	Number
Article not cited by any systematic reviews or meta‐analyses	195
Article cited in a review where no quality assessment was done	77
Article cited in a review where quality assessment was done, but RoB 2, CONSORT, ROBIS or PRISMA were not used	44
Article cited in a review where one or more of the above tools were used, but the article's quality was not assessed	6
Article cited in a review where one or more of the above tools were used AND the article's quality was assessed	0
Article not found in Web of Science database	49

## APPENDIX D COMMONLY AND RARELY REPORTED CONSORT, PRISMA ITEMS

**Commonly reported CONSORT items (by** >**95% of studies)**

**Table:**

2a. Scientific background and explanation of rationale	
Yes	82 (100%)
No	0 (0%)
2b. Specific objectives or hypotheses	
Yes	82 (100%)
No	0 (0%)
4a. Eligibility criteria for participants	
Yes	82 (100%)
No	0 (0%)
12a. Statistical methods used to compare groups for primary and secondary outcomes	
Yes	79 (96.3%)
No	3 (3.7%)
22. Interpretation	
Yes	80 (97.6%)
No	2 (2.4%)

**Rarely reported CONSORT items (by** <**5% of studies)**

**Table:**

6b. Any changes to trial outcomes after the trial commenced, with reasons	
Yes	0 (0%)
No	82 (100%)

**Commonly reported PRISMA items (by** >**95% of studies)**

**Table:**

3. Rationale	
Yes	276 (95.5%)
No	13 (4.5%)
5. Eligibility criteria	
Yes	276 (95.5%)
No	13 (4.5%)
23a. Provide a general interpretation of the results	
Yes	279 (96.5%)
No	9 (3.1%)
23d. Discuss implications of the results for practice, policy, and future research.	
Yes	282 (97.6%)
No	6 (2.1%)

**Rarely reported PRISMA items (by** <**5% of studies)**

**Table:**

2. Structured summary	
Yes	0 (0.0%)
No	289 (100.0%)
10b. Data items‐ define study characteristics	
Yes	5 (1.7%)
No	284 (98.3%)
16b. Study selection‐cite excluded studies	
Yes	14 (4.8%)
No	274 (94.8%)

## APPENDIX E RESULTS OF STATISTICAL ANALYSES

Correlations between study characteristics and CONSORT score (for RCTs):

**Table:**

Variable	Estimate	LCI	UCI
Affiliation to hospital: Yes	−1.695	−3.325	−0.066
Journal impact factor	0.337	0.190	0.484
Published protocol: Yes	4.487	2.920	6.053
Funding type: Other	3.051	0.834	5.269

Correlations between study characteristics and risk of bias (for RCTs):

**Table:**

Variable	OR	LCI	UCI
Number of authors	0.844	0.707	0.976

Correlations between study characteristics and PRISMA score (for systematic reviews):

**Table:**

Variable	Estimate	LCI	UCI
Review type: Cochrane	8.577	3.782	13.373
Published protocol: Yes	5.885	4.667	7.102
Funding type: No funding	1.213	0.033	2.394
Funding type: Government	3.002	0.828	5.176
Conflict of interest: Present	4.282	1.757	6.807
Age range reported: Yes	−1.324	−2.401	−0.246

Correlations between study characteristics and risk of bias (for systematic reviews)

**Table:**

Variable	OR	LCI	UCI
Published Protocol: Yes	0.024	0.002	0.154
